# An Unconventional Regimen of Carboplatin and Paclitaxel in Metastatic Colorectal Carcinosarcoma: A Case Report and Review of Literature

**DOI:** 10.3390/curroncol30050369

**Published:** 2023-05-10

**Authors:** Changsu Lawrence Park, Charles Vincent Rajadurai, Tuyet Nhung Ton Nu, Victoria Mandilaras

**Affiliations:** 1 Department of Medical Oncology, McGill University Health Center, McGill University, Montreal, QC H4A 3J1, Canada; changsu.park@mail.mcgill.ca (C.L.P.);; 2 Department of Pathology, McGill University Health Center, McGill University, Montreal, QC H4A 3J1, Canada

**Keywords:** carcinosarcoma, metastatic, colorectal cancer, systemic treatment

## Abstract

Colorectal carcinosarcoma is an exceedingly rare subtype of colorectal cancer that displays the histological and molecular features of both mesenchymal and epithelial tumors. Due to its rarity, there are no guidelines regarding the systemic treatment of this disease. This report describes a case of a 76-year-old woman with colorectal carcinosarcoma with extensive metastatic burden treated with carboplatin and paclitaxel. After four cycles of chemotherapy, the patient had an excellent clinical and radiographical response to treatment. To the best of our knowledge, this is the first report addressing the use of carboplatin and paclitaxel in this disease. We reviewed seven published case reports of metastatic colorectal carcinosarcoma where various systemic treatments were offered. Remarkably, there are no previously published reports where even a partial response was noted, which underscores the aggressiveness of this disease. While further studies are required to validate our experience and assess long-term outcomes, this case suggests an alternative treatment regimen for metastatic colorectal carcinosarcoma.

## 1. Introduction

Solid malignancies are largely divided into carcinoma and sarcoma based on their epithelial and mesenchymal tissue of origin, respectively. Carcinosarcoma (also described as sarcomatoid carcinoma or spindle cell carcinoma) is a rare subcategory of cancer that displays the histological and molecular features of both mesenchymal and epithelial tumors [1]. The pathogenesis of carcinosarcomas is not fully understood but molecular profiling suggests a monoclonal origin with late divergence and transformation to its biphasic state [2]. Carcinosarcomas have been reported to arise from various organs; a predominance is seen in the gynecologic tract [3], respiratory tract [4], and head and neck [5].

Colorectal cancer is the third leading cause of cancer-related deaths and fourth most diagnosed cancer worldwide [6]. Carcinosarcoma of lower gastrointestinal tract origin (hereon referred to as colorectal carcinosarcoma) nevertheless remains an exceedingly rare subtype of colorectal cancer with less than 40 cases reported in the literature. Similar to colorectal cancer, patients often present with rectal bleeding, abdominal pain, weight loss, and bowel obstruction [7,8]. Based on the limited data available, metastatic sites also follow a similar tropism (i.e., liver, lung, lymph nodes, and peritoneum) [7,8]. Despite these similarities, colorectal carcinosarcoma is associated with a more aggressive phenotype and poorer prognosis. While surgical resection can be performed when feasible, there is no level 1 evidence, guidelines or consensus regarding the systemic treatment of colorectal carcinosarcoma, especially in the metastatic setting. Most case reports describe the use of 5-fluorouracil-based treatments, extrapolating from the treatment of colorectal adenocarcinoma, with variable success [9,10,11,12,13].

This case report describes a patient with metastatic colorectal carcinosarcoma who had an excellent initial clinical and radiological response to carboplatin and paclitaxel, a previously unreported regimen for this disease.

## 2. Case Presentation

A 76-year-old woman presented to the emergency department of a tertiary care center with a two-month history of fatigue, ten-kilogram weight loss, progressive constipation, and abdominal pain. Her past medical and surgical history was remarkable for hypertension, hypothyroidism, appendectomy, and cholecystectomy. She had no history of tobacco use and consumed alcohol occasionally. Computed tomographic scan of the abdomen and pelvis demonstrated a heterogenous solid mass measuring 13.5 × 18.5 × 21.5 cm in the lower abdomen and pelvis, inseparable from multiple bowel loops, uterine serosa and ovaries. Imaging also demonstrated compression of the right ureter causing right-sided moderate hydronephrosis. Biochemistry revealed an elevated creatinine of 137 umol/L (reference 40–85 umol/L) compatible with post-obstructive acute kidney injury, which recovered to 84 umol/L after a double J stent placement.

Staging computed tomography of the chest, abdomen and pelvis revealed multiple infracentrimetric indeterminate pulmonary nodules (largest 5 mm), peritoneal carcinomatosis/omental caking and ill-defined liver lesions, mild bilateral pleural effusion and moderate ascites. At the time of diagnosis, her carcinoembryonic antigen was 61.7 μg/L (reference 0–4.9 μg/L), cancer antigen-125 was 1451 U/mL (reference 0–35 U/mL), cancer antigen-15.3 was 36.6 U/mL (reference 0–31.3 U/mL) and cancer antigen-19.9 was 25.4 U/mL (reference 0–35 U/mL). 

**Figure 1:**
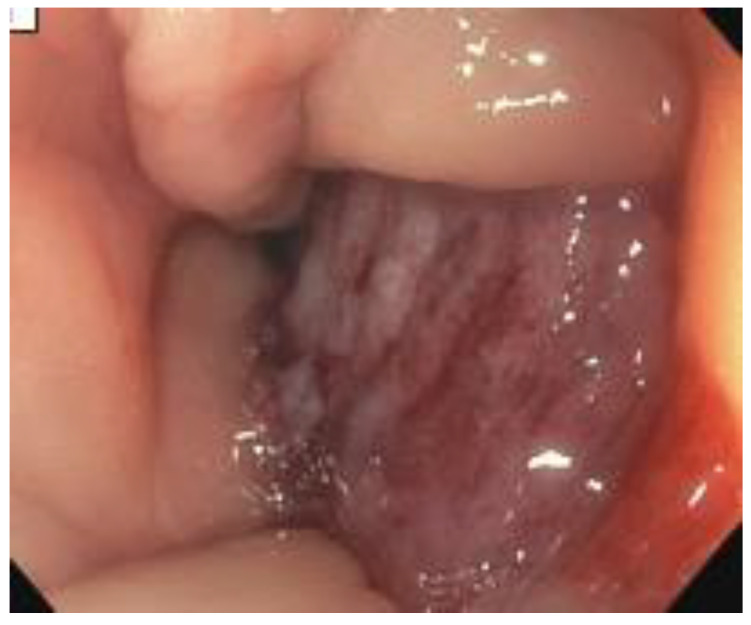
Colonoscopy revealing mass at sigmoid colon.

A colonoscopy was performed, showing an extrinsic bulge at the ascending colon and hemi-circumferential friable tissue in the sigmoid colon suspicious for malignancy (Figure 1). Biopsy of the suspicious sigmoid tissue, however, showed no evidence of malignancy, but this was thought to be due to sampling error. Given the low likelihood that the patient would tolerate another colonoscopy, it was not repeated. The patient also underwent an endocervical exam and biopsy, which was negative for cancer.

**Figure 2:**
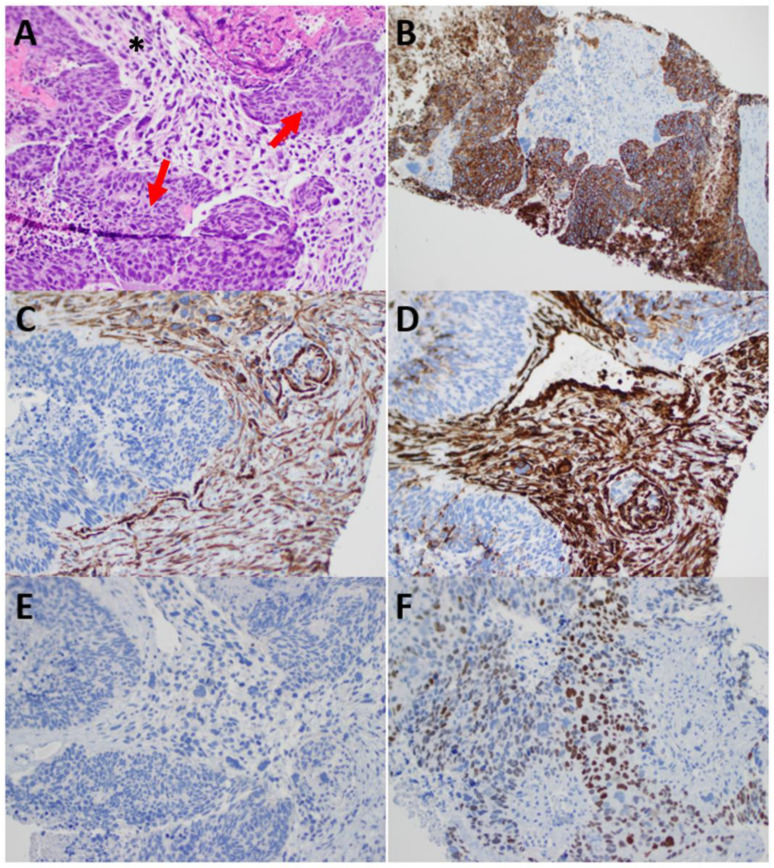
Immunohistochemistry of omental nodule. (**A**): Arrow denotes areas of carcinoma. * denotes areas of sarcoma. (**B**): Cytokeratin (CK) 8/18 staining positive in carcinoma and negative in sarcoma. (**C**): Caldesmon staining positive in sarcoma and negative in carcinoma. (**D**): Vimentin staining positive in sarcoma and negative in carcinoma. (**E**): PAX8, WT1, ER staining negative, which exclude the genital tract origin. (**F**): CDX-2 staining positive, which excludes the genital tract origin and includes the GI tract.

She then underwent an ultrasound guided omental biopsy. On histology, the tumor consisted of carcinomatous (cohesive cells with pleomorphism, hyperchromasia, high N/C ratio, high mitosis, atypical mitosis and apoptotic bodies) and sarcomatous (spindle cells with severe atypia, high N/C ratio, mitosis) components. On immunohistochemistry, the carcinomatous cells stained positive for CK8/18, CK7 and CDX2, negative for Pax8, WT1, CK20, ER and PR. The sarcomatous cells were positive for vimentin, SMA (patchy) and caldesmon; negative for CK 8/18, CK7, CK20. The above IHC profile confirmed a carcinosarcoma (Figure 2). Both components showed intact nuclear expression for mismatch repair proteins (MLH1, MSH2, MSH6 and PMS2) and abnormal (diffuse and strong) P53 and P16. The case was presented at a multidisciplinary tumor board, where a consensus was reached that the pathology was more compatible with gastrointestinal origin than gynecologic origin given the findings on colonoscopy and immunohistochemical positivity for CK7 and CDX2 and negativity for Pax8, WT1,ER and PR. The disease was deemed unresectable, and a consultation was placed to medical oncology for the consideration of systemic treatment.

**Figure 3:**
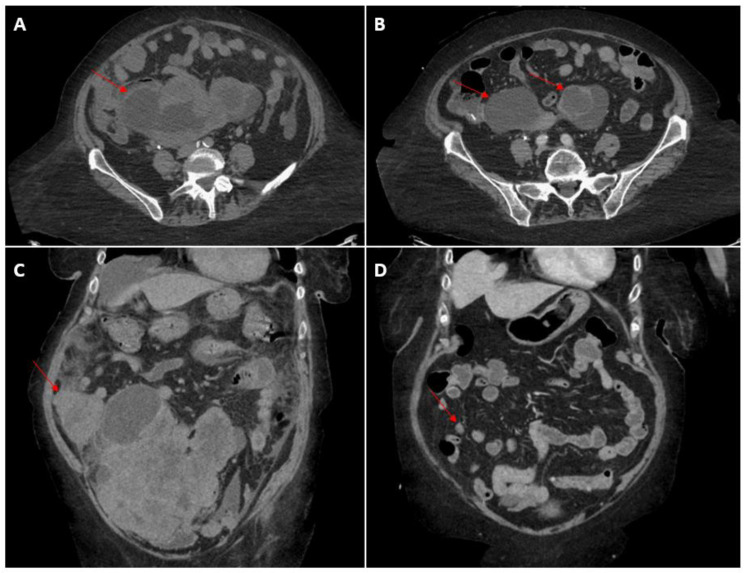
Representative computed tomography images after 4 cycles of chemotherapy. (**A**): pelvic mass on presentation. (**B**): pelvic mass after 4 cycles of carboplatin and paclitaxel. (**C**): right peritoneal implant on presentation. (**D**): right peritoneal implant after 4 cycles of carboplatin and paclitaxel.

Several chemotherapy options were discussed with the patient, including FOLFOX (leucovorin, 5-fluorouracil, oxaliplatin) versus carboplatin and paclitaxel, which is more commonly used with carcinosarcoma of uterine origin. After assessing the risk versus benefits, a shared decision was made to initiate an alternative regimen of dose-reduced carboplatin and paclitaxel every 3 weeks given its relative ease of administration. The patient underwent 4 cycles of paclitaxel (140 mg/m^2^) and carboplatin (AUC 4) uneventfully with excellent tolerance to treatment. Clinical improvement was noted, evidenced by reduced pain, increased exercise tolerance and oral intake, and normalization of her serum creatinine level. Restaging scans after 4 cycles of chemotherapy demonstrated intervals with a decrease in size and enhancement with internal necrosis of the large invasive pelvic mass, measuring approximately 12.9 × 8.8 cm, previously 16.4 × 15.6 cm, decrease in size of liver lesions (Segment 8: 0.9 cm from 1.8 cm; Segment 5: 1.2 cm from 1.6 cm), decrease in the right omental metastatic implant abutting the cecum, measuring 1.2 cm × 1.2 cm, previously 5.1 × 6.7 cm, and interval drastic improvement in peritoneal carcinomatosis, stable lung lesions and resolution of bilateral pleural effusion (Figure 3).

Her fifth cycle was delayed by severe symptomatic COVID-19 infection. The patient was not vaccinated for COVID-19. Upon re-assessment in clinic after her quarantine period of 21 days per hospital policy, her performance status had declined to ECOG 4. Restaging scans 2 months after her last chemotherapy showed disease progression. She was no longer a candidate for further chemotherapy based on her functional status.

## 3. Discussion

This case report describes the use of an unconventional regimen of carboplatin and paclitaxel for the treatment of metastatic colorectal carcinosarcoma. There are currently no guidelines for the treatment of this aggressive malignancy due to the rarity of colorectal carcinosarcoma. As such, building on the shared experience of clinicians through case reports is required to impact its current poor prognosis.

Histologically, carcinosarcomas are defined by the concurrent presence of high-grade adenocarcinoma and sarcoma in the tumour. The two distinct components are theorized to arise from a common progenitor based on molecular studies, but the exact pathogenesis and driver of the divergent phenotypes have not yet been well-characterized [2]. The adenocarcinoma component is known to stain positive for epithelial markers and various patterns of positive cytokeratin staining have been reported in colorectal carcinosarcoma [9,13,14,15]. The sarcomatous component is often positive for vimentin, SMA and desmin. Our patient displayed the classic immunohistochemical profile of carcinosarcoma. Molecular staining with various markers that are commonly positive in the gastrointestinal tract and gynecologic tract favoured a gastrointestinal origin. This, combined with a large suspicious mass in the sigmoid colon on colonoscopy, allowed us to conclude a diagnosis of metastatic colorectal carcinosarcoma.

To the best of our knowledge, there are seven published case reports of metastatic colorectal carcinosarcoma where systemic therapy was offered to patients (Table 1). Five of these studies used the conventional 5-flurouracil backbone chemotherapy, which is the standard of care for metastatic colorectal adenocarcinoma. Of these, only one case resulted in a positive clinical response of stable disease of two years [13]. Macaigne et al. reported the use of doxorubicin, a commonly used chemotherapy regimen for the treatment of sarcoma, which did not yield a positive clinical outcome [14]. Patel et al. described the use of gemcitabine and docetaxel, but follow-up to this regimen was not reported [15]. Throughout this literature review, it is evident that there is a paucity of data available to inform clinicians of the best treatment for colorectal carcinosarcoma, reaffirming the poor prognosis associated with this rare disease.

The combination of carboplatin and paclitaxel is a comparatively well-studied regimen for treatment of the relatively more common uterine carcinosarcoma. While it is premature to extrapolate these data to the colorectal setting, it is at least worth considering if this regimen could be effective for targeting a common pathway in the pathogenesis of all carcinosarcomas irrespective of the primary organ—similar to the use of platinum-based chemotherapy for small cell carcinomas notwithstanding the primary site. Carboplatin and paclitaxel were offered to our patient through shared decision-making given her extensive metastatic burden and the poor likelihood of the patient tolerating a 5-flurouracil-based regimen. We observed a significant clinical and radiographical response, which, to our knowledge, is the first to be reported in the literature for metastatic colorectal carcinosarcoma. Unfortunately, the patient succumbed to COVID-19 infection and a sustained response to chemotherapy could not be observed as treatment was interrupted due to quarantine for COVID-19 and deterioration of performance status unrelated to her cancer.

An area of further clinical investigation that is also required is the use of next generation sequencing in colorectal carcinosarcoma. Various mutations have been studied in metastatic colorectal adenocarcinoma and translated to clinical implications for systemic treatment. For example, mutations within the RAS and BRAF pathway are common in colorectal adenocarcinoma and predict response to the addition of EGFR inhibition to classical chemotherapy [16]. Unfortunately, next generation sequencing was not performed in our patient as the results were unlikely to impact the first-line treatment. Nevertheless, it remains unknown whether specific mutations are found in colorectal carcinosarcoma and if targeted treatment will impact the poor outcome of these patients. To the best of our knowledge, next generation sequencing results yielding targetable mutations in metastatic colorectal carcinosarcoma have not yet been reported in the literature.

Further studies are required to validate our experience and assess the long-term outcomes of the use of carboplatin and paclitaxel for the treatment of metastatic colorectal carcinosarcoma. Nevertheless, this case report suggests an alternative regimen for the treatment of metastatic carcinosarcoma, especially if there are significant contraindications to 5-fluorouracil based chemotherapy.

## Figures and Tables

**Table 1:** Summary of published systemic treatments for metastatic colorectal carcinosarcoma.

Ref.	Metastatic Sites	Surgery?	Systemic Treatment	Best Response
[13]	Liver	Yes	Capecitabine	Stable disease sustained for 2 years
[14]	Liver, peritoneum	Yes	Doxorubicin	Disease progression
[15]	Liver	Yes	Gemcitabine + Docetaxel	Unknown
[9]	Peritoneum	Yes	1: Mitomycin C + 5-FU 2: Cyclophosphamide + doxorubicin + carboplatin	Disease progression
[10]	Liver	Yes	5-FU + Leucovorin	Disease progression
[11]	Liver, Lung, Brain	Yes	5-FU + Leucovorin	Disease progression
[12]	Lung	Yes	FOLFIRI + Bevacizumab	Disease progression
*	Liver, peritoneum	No	Carboplatin + Paclitaxel	Partial response

Abbreviations. 5-FU: 5-fluorouracil. FOLFIRI: 5-FU, Leucovorin, Irinotecan. * Denotes this case.

## Data Availability

Not applicable.
